# Exosomes Derived from Squamous Head and Neck Cancer Promote Cell Survival after Ionizing Radiation

**DOI:** 10.1371/journal.pone.0152213

**Published:** 2016-03-23

**Authors:** Lisa Mutschelknaus, Carsten Peters, Klaudia Winkler, Ramesh Yentrapalli, Theresa Heider, Michael John Atkinson, Simone Moertl

**Affiliations:** 1 Helmholtz Zentrum München, German Research Center for Environmental Health, Institute of Radiation Biology, Neuherberg, Germany; 2 Department of Chemistry, Technical University of Munich, Munich, Germany; 3 Radiation Biology, Technical University of Munich, Munich, Germany; Gustave Roussy, FRANCE

## Abstract

Exosomes are nanometer-sized extracellular vesicles that are believed to function as intercellular communicators. Here, we report that exosomes are able to modify the radiation response of the head and neck cancer cell lines BHY and FaDu. Exosomes were isolated from the conditioned medium of irradiated as well as non-irradiated head and neck cancer cells by serial centrifugation. Quantification using NanoSight technology indicated an increased exosome release from irradiated compared to non-irradiated cells 24 hours after treatment. To test whether the released exosomes influence the radiation response of other cells the exosomes were transferred to non-irradiated and irradiated recipient cells. We found an enhanced uptake of exosomes isolated from both irradiated and non-irradiated cells by irradiated recipient cells compared to non-irradiated recipient cells. Functional analyses by exosome transfer indicated that all exosomes (from non-irradiated and irradiated donor cells) increase the proliferation of non-irradiated recipient cells and the survival of irradiated recipient cells. The survival-promoting effects are more pronounced when exosomes isolated from irradiated compared to non-irradiated donor cells are transferred. A possible mechanism for the increased survival after irradiation could be the increase in DNA double-strand break repair monitored at 6, 8 and 10 h after the transfer of exosomes isolated from irradiated cells. This is abrogated by the destabilization of the exosomes. Our results demonstrate that radiation influences both the abundance and action of exosomes on recipient cells. Exosomes transmit prosurvival effects by promoting the proliferation and radioresistance of head and neck cancer cells. Taken together, this study indicates a functional role of exosomes in the response of tumor cells to radiation exposure within a therapeutic dose range and encourages that exosomes are useful objects of study for a better understanding of tumor radiation response.

## 1 Introduction

Exosomes are a subclass of extracellular microvesicles that are secreted by most cell types, including tumor cells. They are endocytic in origin and released into the extracellular environment through fusion of cytosolic multivesicular bodies with the plasma membrane. Exosome cargo includes a wide range of proteins, mRNAs, microRNAs and long non-coding RNAs [1–4]. Functional studies reveal that exosomes act as extracellular communicators by delivering their content to a target cell via membrane fusion, or alternatively by endocytosis [5]. In 2007 Valadi et al. demonstrated that exosomes are able to shuttle RNA between cells. The transfer of murine mast cell exosomes to human mast cells results in the translation of murine mRNA, proving that the delivered RNA molecules are functional in the recipient cells [3].

Absorbed exosomes are able to modify biological functions of the recipient cells, where they may confer a new phenotype, such as metastasis [6], angiogenesis [7] and migration [8]. The exosomal composition of the extracellular milieu is modified by cellular stressors, leading to changed, mostly protective effects upon recipient cells. Thus exosomes derived from cells exposed to oxidative stress provide resistance against oxidative stress to non-exposed recipient cells [9]. In breast cancer cell lines, hypoxia also increases the release of exosomes carrying increased amounts of miR-210. This enhances survival and invasion of recipient cells [10]. In the context of ionizing radiation exosomes derived from irradiated glioma cells enhance the migration of recipient glioma cells [11]. Exosomes may thus influence communication of radiation effects between non-targeted and targeted cells (bystander-like signaling), such as genomic instability [12–14].

Squamous cell carcinomas are common malignancies of the head and neck region. Radiochemotherapy or radiotherapy is the most common therapy for HNSCC (head and neck squamous cell carcinoma) patients with locally advanced and unresectable tumors [15]. However, therapy resistance and tumor recurrence pose a major challenge and their mechanisms are not well understood. Since exosomes are emerging players in drug resistance we aim to evaluate whether exosomes could affect the radiation response of head and neck squamous carcinoma cells [16–19]. For this purpose we determined the impact of ionizing radiation within a moderate dose range on exosome release and uptake in HNSCC. In order to analyze a putative functional role of exosomes we added exosomes isolated from differentially irradiated donor cells, and analyzed resulting effects on proliferation, survival and DNA repair of recipient HNSCC after a treatment with ionizing radiation.

## 2 Materials and Methods

### 2.1 Cell culture and irradiation

Head and neck cancer cell lines BHY (DSMZ no.: ACC 404) and FaDu (ATCC^®^HTB43^™^) were incubated at 37°C and a relative air humidity of 95%. BHY cells were cultivated in high Glucose DMEM culture medium (Dulbecco’s modified Eagle’s medium, Gibco) plus 10% fetal calf serum (Bio&SELL), 2 mM L-Glutamine and sodium pyruvate at 10% CO_2_. FaDu cells were maintained in low Glucose DMEM (GE Healthcare) supplemented with 10% fetal calf serum, 2 mM L-Glutamine and 25 mM HEPES at 5% CO_2_. Cell line identities were validated by sequencing of nine different loci: D5S818, D13S317, D7S820, D16S539, VWA, TH01, AM, TPOX, CSF1PO (performed by Eurofins Genomics, S1 and S2 Tables). A mycoplasma screening revealed negative results.

Cells were irradiated with γ-rays emitted by a ^137^caesium source at the irradiation facility HWM-D2000 (Wälischmiller Engineering) with a dose rate of 1 Gy per 2.04 minutes.

### 2.2 Isolation of exosomes

**Fig 1:**
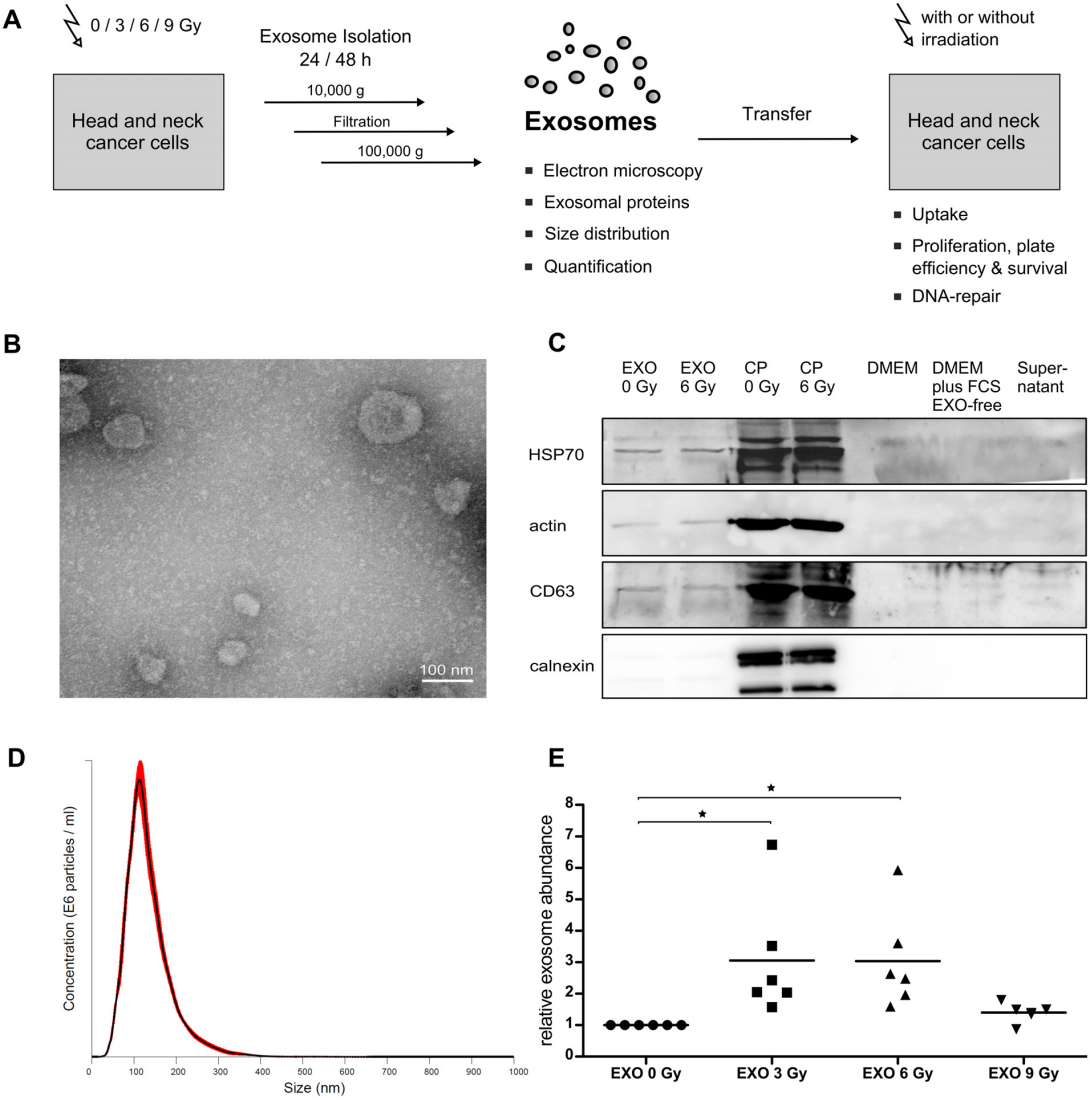
Characterization of isolated exosomes. **(A)** Scheme of exosome analysis. **(B)** Transmission electron micrograph showing exosomes isolated from the cell culture supernatant of 3 Gy-irradiated BHY cells [scale bar: 100 nm]. **(C)** Representative immunoblot of HSP70, actin, CD63 and calnexin performed with exosome lysates (EXO) and cell lysates (CP) harvested 24 hours after irradiation. DMEM medium, DMEM medium supplemented with exosome-depleted fetal calf serum as well as supernatant after ultracentrifugation were loaded as controls. **(D)** Size distribution of exosomes from non-irradiated BHY cells measured with NanoSight technology. **(E)** Relative exosome abundance of BHY exosomes isolated 24 hours after irradiation with 0, 3, 6 and 9 Gy [n = 6, p-value < 0.05].

For the isolation of exosomes the protocol of Théry et al. was adapted [20] (Fig 1A). 5 × 10^5^ cells were seeded per 10-cm petri dish, 72 hours later the medium was replaced by 8 ml exosome-free medium and cells were irradiated over a moderate dose range of 0–9 Gy. Exosomes isolated from non-irradiated cells received the abbreviation EXO 0 Gy, while exosomes from irradiated cells were named EXO 3 Gy, EXO 6 and EXO 9 Gy. Exosome isolation was conducted from the conditioned medium collected 24 and 48 hours after irradiation. To eliminate detached cells, dead cells as well as cellular fragments, the cell culture supernatant was centrifuged with 10,000 g for 30 minutes and afterwards passed through a filter with a pore size of 0.22 μm. An ultracentrifugation step with 100,000 g enabled the sedimentation of the exosomes (75 minutes, 4°C). The supernatant was discarded and the exosomal pellet was resuspended in 2 ml PBS. After repetition of another ultracentrifugation step (100,000 g) the supernatant was discarded and the exosomes were resuspended in PBS. Exosomal preparations were stored at -20°C. Exosome donor cells were harvested 24 and 48 hours after irradiation using a cell scraper. After washing the cellular pellet twice with PBS, the pellet was frozen at -20°C. For the preparation of exosome-free medium, bovine exosomes were removed from fetal calf serum by centrifugation at 100,000 g for 14 hours.

### 2.3 Electron microscopy of exosomes

Undiluted sample (isolated from 3 ml conditioned medium) was absorbed onto glow discharged carbon coated grids (G2400C from Plano) for 2 minutes. The solution was blotted of and negatively stained with 4% ammonium molybdate (Sigma-Aldrich) solution for 30 seconds. Micrographs were recorded with a Jeol JEM 100CX electron microscope at 100 kV onto Kodak SO163 film. Negatives were digitized with a Hasselblad Flextight X5 scanner at 3000 dpi, resulting in a pixel size of 0.25 nm/px. For visualization images were binned to 1 nm/px.

### 2.4 Exosome quantification and determination of exosomal size-distribution

Exosome amount and size distribution was analyzed by using the NanoSight LM10 (Malvern) microscope. Exosome preparations (isolated from 5 ml conditioned medium) were diluted 1:100 to 1:2000 with H_2_O to achieve 15 to 50 particles per frame for tracking. Samples were each analyzed three times for 30 seconds.

### 2.5 Exosome uptake

Exosomes (isolated from 15 ml conditioned medium) were stained with the green fluorescent dye PKH67 (MINI67-1KT, SIGMA-Aldrich Chemie). For this purpose 50 μl of exosome solution were resuspended in 250 μl of the diluent C plus 1.5 μl of the dye (1 mM). After 10 minutes of incubation at room temperature excessive dye was removed by using Exosome Spin Columns (Invitrogen) according to the manufacturer’s protocol. As control an equal amount of dye in diluent C plus 50 μl of PBS was processed similar to exosomes (exosome negative control, -EXO).

To measure the uptake of exosomes 50,000 cells in 200 μl medium were seeded in 48 well plates. After 24 hours equal amounts of PKH67-stained exosomes were added to irradiated and non-irradiated recipient cells. After an additional 3, 6, 8, 10 and 24 hours cells were washed three times with PBS, trypsinized and resuspended in 500 μl of PBS. Uptake was measured on a FACSCAN LSRII (Becton-Dickinson, excitation = 490 nm, emission = 502 nm). For fluorescence microscopy cells were washed three times with PBS fixed with 4% paraformaldehyde washed again with PBS and covered with Vectashield^®^ including Hoechst 33342 for nuclei staining. Pictures were taken with the fluorescence microscope BZ-9000 from Keyence.

### 2.6 Incubation of recipient cells with exosomes

To determine the biological activity of exosomes (proliferation, survival and DSB repair) we incubated the recipient cells with exosomes isolated from identical numbers of donor cells. The exosomes were recovered into volumes to give a three-fold concentration of exosomes compared to the native conditions.

### 2.7 Proliferation and clonogenic survival after transfer of exosomes

The effect of exosomes on the proliferation was determined with the Presto Blue^™^ Cell Viability Reagent Protocol (Life Technologies). 500 or 1500 cells per well were seeded into 96 well plates in 100 μl exosome-free medium. After 24 hours exosomes (isolated from 300 μl conditioned medium) were added and the cells were incubated for another 72 hours. For the measurement of cell proliferation 10 μl Presto Blue reagent were added per well, incubated for 40 min at 37°C and fluorescence was determined (Excitation 560 nm; Emission: 590 nm) in a plate reader (Tecan).

For survival determination, a clonogenic survival assay was performed. Cells were seeded in 12 well plates and sham treated or irradiated with 1, 2, 3, 6 and 10 Gy. Immediately afterwards, exosomes (from 2.5 ml conditioned medium) were transferred on the cells which were then incubated for 5 days to allow colony formation from single cells. Subsequently cells were washed twice with PBS, fixed with 100% ethanol (30 minutes) and finally stained with Giemsa solution (Boehringer Ingelheim, 1:20 in PBS, 30 minutes). Excessive dye was removed and colonies with more than 30 cells were counted.

### 2.8 Detection of DNA double-strand breaks after transfer of exosomes

1,000 to 6,000 cells were seeded in 96 well plates. After reaching a confluence of 50–70% the medium was replaced by 100 μl of exosome-free medium, the cells were immediately irradiated with 2 Gy and exosomes isolated from 300 μl conditioned medium were added. After an incubation of 1, 6, 8 or 10 hours at 37°C the number of DNA DSBs was determined by 53BP1 staining. A fixation step with 4% paraformaldehyde was followed by a permeabilization with 0.2% Triton X-100. Subsequently the cells were blocked with PBS + (1% bovine serum albumin, 0.15% glycine) for 60 minutes and incubated overnight with the primary antibody 53BP1 (dilution 1:500, NB100-305, Novus Biologicals) at 4°C. On the following day the cells were incubated with the secondary antibodies goat anti-rabbit Alexa-488 (dilution 1:200, A-11034, Life Technologies) and sheep anti-mouse Cy-3 (dilution 1:500, 016-160-084, Jackson Lab) for 1 hour. Nuclei were stained with Hoechst 33342 (SIGMA-Aldrich Chemie) and the cells were covered with Vectashield^®^ Mounting Medium (Linaris). Analysis was performed with the fluorescence microscope Biorevo BZ-9000 (Keyence). For all experimental conditions the exposure times were maintained and the foci number of 60 cells per condition was determined.

### 2.9 Validation of exosomal stability

To test the stability, exosomes were incubated for 30 minutes at 37°C either with RNase A from Qiagen (5 μg/μl or 400 μg/μl) or a detergent-peptidase-mixture (0.2% Triton X-100/Trypsin, 2:1). Then the exosomes were used in DNA repair assays as described above.

### 2.10 Protein analysis

Cells were lysed in lysis buffer II (25 mM Tris pH 7.5, 120 mM NaCl, 1% Triton X-100, 1% PSMF, 1 mM NOV, 1 mM Leupeptin) for 1 hour on ice. After centrifugation the protein concentration of the collected supernatants was determined by applying the BCA-assay using bovine serum albumin as standard (Pierce^™^ BCA Protein Assay Kit, Thermo Fisher Scientific).

Western blot analysis was accomplished according to standard procedures using 10 μg of cellular protein and a volume of 12 μl exosome lysate corresponding to the exosome amount in 30 ml conditioned medium for SDS polyacrylamide gel electrophoresis. Separated proteins were blotted on nitrocellulose membranes and incubated with primary antibodies directed against CD63 (sc15363, SantaCruz), HSP70 (MA3-007, Affinity Bioreagents), actin (SAB1305567, SIGMA-Aldrich Chemie) and calnexin (sc11397, SantaCruz). Horseradish peroxidase-conjugated anti-rabbit and anti-mouse antibodies (sc2004 and sc2005, SantaCruz) were used to detect antigen antibody binding via chemoluminescence (Amersham ECL detection kit, GE Healthcare).

### 2.11 Statistical analysis

Data represent the mean of independent, biological replicates ± standard deviation (SD). Significance of n-fold changes was calculated by using the paired t-test. To compare means of three or more variables the two-sided ANOVA was applied. For all statistical analysis p < 0.05 was considered statistically significant and p < 0.01 and p < 0.001 was deemed highly significant.

## 3 Results

### 3.1 Radiation increases exosome release from head and neck cancer cells

Exosomes released by the head and neck tumor cell line BHY were isolated by differential ultra-centrifugation. To validate the isolation method exosomes were visualized by transmission electron microscopy. The representative micrograph showed round, cup-shaped structures with a diameter of 30–100 nm (Fig 1B). For further verification of the exosome identity the exosomal marker proteins HSP70, actin and CD63 were detected by western blot in BHY exosomes as well as in lysates of BHY cells. No detectable proteins were present in unused culture medium, in unused medium supplemented with exosome-depleted fetal calf serum or in the supernatant of conditioned medium after ultracentrifugation. The absence of calnexin in the exosome lysates demonstrates that exosome preparations were not contaminated with cell membranes derived from apoptotic bodies or dead cells (Fig 1C). Furthermore the size distribution and the number of isolated exosomes were quantified in six independent preparations for each treatment using NanoSight technology. This approach confirmed a homogenous exosome preparation with an average size of 111–124 nm (n = 6) for the exosomes isolated from either irradiated or non-irradiated cells (Fig 1D). The NanoSight measurement also showed an increase in the number of exosomes recovered from irradiated (EXO 3 Gy, EXO 6 Gy) compared to non-irradiated (EXO 0 Gy) cells 24 hours after irradiation (Fig 1E).

### 3.2 Radiation increases the uptake of exosomes by recipient cells

We compared uptake kinetics of exosomes isolated from irradiated and non-irradiated donor cells as well as their uptake by irradiated and non-irradiated recipient cells. Therefore cells were co-cultured with PKH67-labeled exosomes and exosome uptake was followed by fluorescence microscopy and flow cytometry.

**Fig 2:**
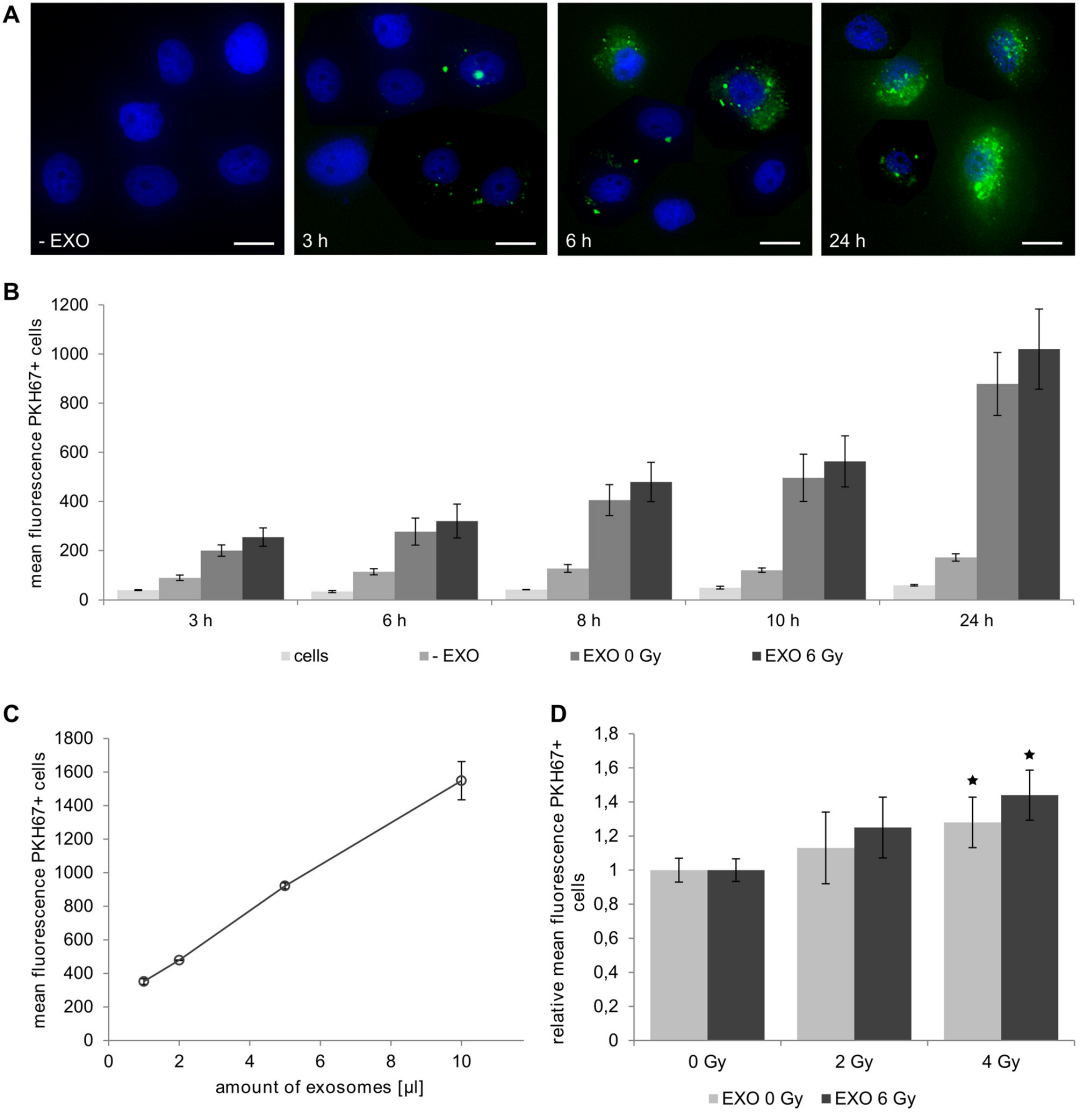
Uptake of exosomes by recipient cells. PKH67-labeled exosomes isolated from irradiated and non-irradiated BHY cells were co-cultivated with BHY cells. **(A)** Representative fluorescence microscopy images for exosome uptake after 3, 6 and 24 hours incubation. Exosomes were stained in green and nuclei were stained blue with Hoechst 33342. **(B)** Uptake of exosomes isolated from 6 Gy-irradiated (EXO 6 Gy) and non-irradiated BHY cells (EXO 0 Gy) after 3, 6, 8, 10 and 24 hours incubation. Mean fluorescence of untreated cells and cells after incubation with stained exosomes or an exosome-negative control (-EXO) is shown (n = 3). **(C)** Dependency of exosomal uptake was determined after 24 hours by using a serial dilution of an exosome preparation. **(D)** Uptake of labeled exosomes by 0, 2 and 4 Gy-irradiated recipient cells after 24 hours. In all experiments a minimum of 10,000 cells were analyzed for each sample [n ≥ 3, ± SD, p-value < 0.05].

Fluorescence microscopy revealed a time dependent uptake of exosomes. After 3 hours clusters of labelled exosomes began to accumulate along cell membranes. Increasing numbers of exosomes attached over time and caused diffuse cytoplasm labelling, which implied an internalization and breakup of exosomes (Fig 2A).

We also quantified the exosome uptake by flow cytometry. The obtained results confirmed our previous microscopy observations and showed that the uptake of exosomes was time dependent (Fig 2B) and linear with the added number of exosomes (Fig 2C). The effect of radiation on the uptake of exosomes by recipient cells was investigated by comparing the uptake kinetics of exosomes isolated from non-irradiated cells to those exosomes isolated from irradiated cells. Fig 2B shows that there was no significant difference in the kinetics between the uptake of exosomes derived from irradiated or non-irradiated donor cells. There was, however, a dose-dependent increase of the uptake of exosomes by irradiated recipient cells compared to that by non-irradiated cells. Thus, exosomal uptake was significantly increased 1.3-fold for exosomes derived from non-irradiated cells and 1.4-fold for exosomes from irradiated donor cells if they were incubated for 24 hours with irradiated recipient cells (4 Gy) compared to uptake by non-irradiated recipient cells (Fig 2D). Taken together these results showed that exosome uptake by recipient cells was time and concentration dependent and that irradiation of recipient cells increased their ability to take up exosomes.

### 3.3 Exosomes from either non-irradiated or irradiated cells increase survival of recipient cells

**Fig 3:**
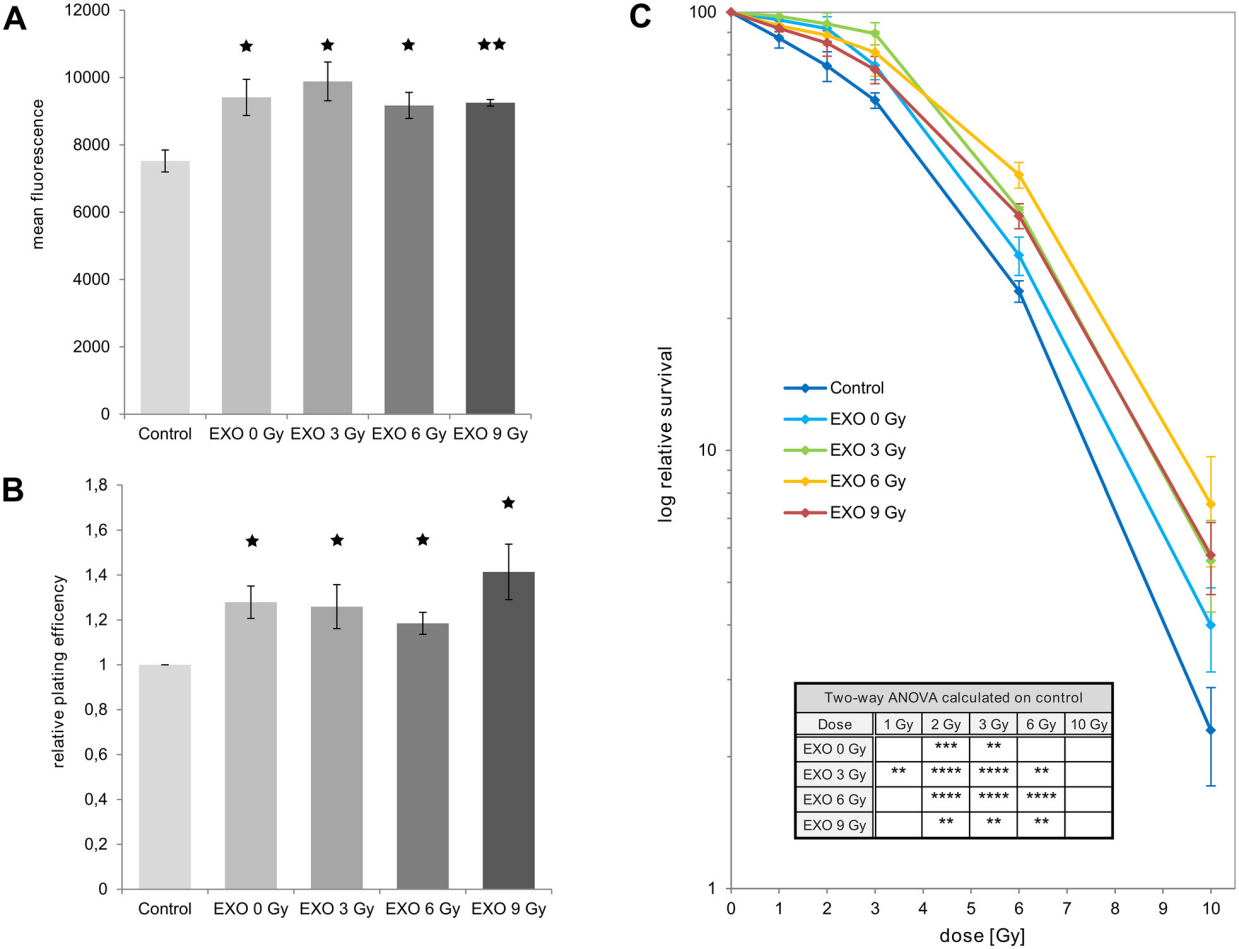
Exosomes affect proliferation, colony formation and clonogenic survival. **(A)** Proliferation of cells cultivated for 3 days in medium containing exosomes isolated from irradiated or non-irradiated cells. As a control an equal amount of PBS without exosomes was added to the recipient cells. **(B)** Plating efficiency of cells cultivated for 5 days in medium containing exosomes isolated from irradiated or non-irradiated cells. As a control an equal amount of PBS without exosomes was added to the recipient cells. **(C)** Clonogenic survival of BHY cells co-cultivated with exosomes isolated from irradiated or non-irradiated cells and control cells (BHY + PBS) were incubated for 5 days after irradiation with the indicated doses [n = 3, ± SD, p-value: * if p < 0.05, ** if p < 0.01 and **** if p < 0.0001].

We were interested whether exosomes from irradiated cells exhibit the same biological effects in the recipient cells as exosomes from non-irradiated cells. To address this question we added exosomes isolated from donor cells irradiated with 0, 3, 6 and 9 Gy to non-irradiated recipient cells and measured cell proliferation. BHY cells treated with exosomes showed greater proliferation than cells cultivated without exosomes (Fig 3A). Accordingly the plating efficiency in the colony formation assay is greater for cells grown with exosomes than for cells grown without exosomes (Fig 3B). However, no significant difference was detected between treatments with exosomes isolated from 0, 3, 6 or 9 Gy-irradiated cells (Fig 3A).

Next the influence of exosomes on the radiation sensitivity of BHY cells was analyzed. Cells were incubated with exosomes, then irradiated with doses of up to 10 Gy and incubated for 5 days. Subsequently, the clonogenic survival was determined. In accordance with the observed proliferation-stimulating effect of exosomes on non-irradiated recipient cells (Fig 3A and 3B) the survival of irradiated recipient cells was increased by the addition of exosomes (Fig 3C and S3 Table). Here, the exosomes isolated from cells irradiated with 6 Gy induced a greater level of radiation resistance than exosomes from non-irradiated cells (Fig 3B). These results suggest that exosomes from BHY cells generally support proliferation and radiation resistance.

### 3.4 Exosomes affect rates of DNA double-strand break repair

**Fig 4:**
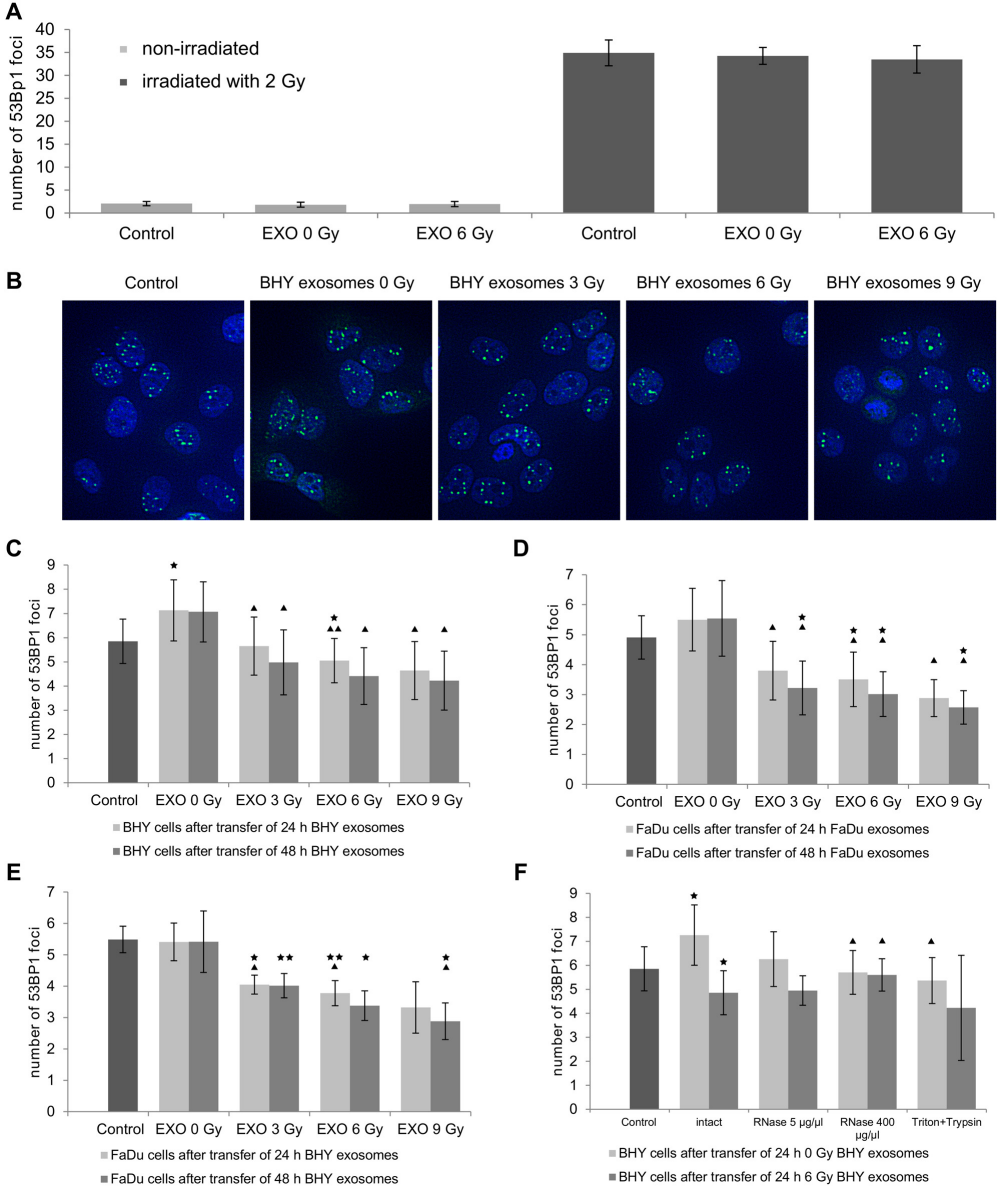
Exosomes modulate the repair of DNA DSBs in irradiated recipient cells. **(A)** Number of 53BP1 foci in BHY cells 1 hour after irradiation with 0 and 2 Gy and transfer of BHY exosomes isolated 24 hours after irradiation with 0 and 6 Gy [n = 5]. **(B)** Representative images of 53BP1 foci in BHY cells 6 hours after 2 Gy and transfer of BHY exosomes isolated 24 hours after irradiation with 0, 3, 6 or 9 Gy (53BP1 foci green, nuclei blue). **(C)** Number of 53BP1 foci in BHY cells 6 hours after 2 Gy and transfer of BHY exosomes isolated 24 and 48 hours after irradiation [n_1_ (control; EXO 0 Gy 24 h; EXO 6 Gy 24 h) = 6, n_2_ (EXO 0 Gy 48 h; EXO 3 Gy; EXO 6 Gy 48 h; EXO 9 Gy) = 3]. **(D)** Number of 53BP1 foci in FaDu cells 6 hours after 2 Gy and transfer of FaDu exosomes [n = 3]. **(E)** Number of 53BP1 foci in FaDu cells 6 hours after 2 Gy and transfer of BHY exosomes [n = 3]. **(F)** Number of 53BP1 in BHY cells after 2 Gy and transfer of destabilized BHY exosomes. Exosomes from BHY cells isolated 24 hours after irradiation with 0 and 6 Gy were treated with RNase A or a mixture of Triton and Trypsin [n_1_ (control; intact) = 6; n_2_ (RNase A 5 μg/μl) = 2; n_3_ (RNase A 400 μg/μl; Triton + Trypsin) = 3]. For all experiments the ± SD was shown and p-values calculated on control were considered to be significant if * p < 0.05 and highly significant ** if p < 0.01, while ^▲^ p < 0.05 and ^▲▲^ p < 0.01 indicate significant differences to EXO 0 Gy.

Since Dutta et al. showed that exosomes released from breast cancer cells can alter the phosphorylation status of DNA damage repair proteins [21], we analyzed the rate of DNA double-strand break (DSB) repair in irradiated recipient cells to elucidate the mechanism for the increased survival of cells after addition of exosomes. Exosomes from irradiated and non-irradiated BHY cells were transferred to irradiated BHY cells (2 Gy) and the number of DNA DSB foci was analyzed after 1 and 6 hours. Quantification of DNA DSB repair foci 1 hour after radiation exposure revealed no difference in the number of induced foci between control cells and cells incubated with exosomes either from non-irradiated or from irradiated donor cells (Fig 4A). Six hours after treatment we found a decreased number of repair foci in BHY cells incubated with exosomes isolated 24 hours after irradiation of BHY cells when compared to cells incubated with exosomes from non-irradiated BHY cells, suggesting a quicker rate of repair (Fig 4B and 4C). Similar effects were seen after 6 hours for exosomes isolated 48 hours after irradiation (Fig 4C). Also the analysis of the distribution of foci numbers per cells after incubation with exosomes reflected the increased repair in cells treated with exosomes from irradiated donor cells. Especially the number of cells with high foci number (> 12) is decreased after incubation with EXO 6 Gy (S1A Fig). Moreover the observed effects were also present 8 and 10 hours after irradiation (S1B Fig). If the cells were pre-incubated for 24 hours with the exosomes, then irradiated with 2 Gy and fixed after 6 hours, the quicker repair induced by exosomes from irradiated donor cells was still observable (S1B Fig). An addition of exosomes from non-irradiated BHY cells appeared to slightly increase the foci number in comparison to the control (PBS) in the recipient cells 6 hours after irradiation (Fig 4B and 4C). This effect was not present after the pre-incubation of cells with exosomes and subsequent irradiation (S1C Fig).

Exosome-stimulated DNA repair was confirmed using a second head and neck cancer cell line FaDu. Again the incubation of FaDu recipient cells with exosomes isolated from irradiated FaDu cells decreased the amount of DNA repair foci (Fig 4D). To test the cell type specificity of exosome-induced effects we added exosomes isolated from BHY cells to irradiated FaDu cells. Exosomes from BHY cells are able to execute similar radioprotective effects on FaDu cells (Fig 4E).

Finally we destabilized exosomes through high concentration RNase A treatment or by adding a detergent-peptidase-mixture. Destabilized 0 Gy and 6 Gy exosomes were unable to change the number of repair foci in comparison to untreated exosomes indicating a loss of function due to the treatment (Fig 4F). Summarizing these results, exosomes influence the repair of DNA DSBs in a dose dependent, cell type unspecific manner.

## 4 Discussion

Cell communication via exosomes is able to influence the fate of cells in stress situations [9, 10, 22, 23]. We now show a contribution of exosomes to the increased survival of head and neck cancer cells after irradiation. Exosomes secreted within 24 hours after irradiation have an impact on proliferation, cell survival, and DNA repair efficiency. As a consequence, cell communication via exosomes during anti-tumor radiation may promote resistance of cancer cells and enhance survival of head and neck cancer cells both, in and outside of the radiation field. Therefore, a better understanding of the underlying mechanisms of exosomes in the radiation response will be needed to improve strategies for radiation therapy.

### 4.1 Exosomes increase survival and proliferation of head and neck squamous carcinoma cells

We show that exosomes influence the fate of irradiated and non-irradiated BHY and FaDu head and neck cancer cells. Exosomes increase the survival of irradiated recipient cells. The prosurvival effects of exosomes from irradiated donor cells were more pronounced than those induced by exosomes from non-irradiated donor cells. In accordance Hazawa et al. showed that the transfer of exosomes from non-irradiated cells to 8 Gy-irradiated mesenchymal stem cells results in increased survival [24].

Correspondingly, exosomes induce proliferation in non-irradiated recipient cells. This effect is independent of radiation-treatment of the exosome donor cells. In the recent literature the effects of exosomes on proliferation are discussed controversially. Similar to our results, exosomes derived from bladder cancer cells, chronic myeloid leukemia cells, or mast cells increase the proliferation of recipient cells after exosome transfer [8, 25–27]. However, Jella et al. showed reduced viability of keratinocytes after incubation in exosome-containing culture medium [14].

### 4.2 Exosomes affect the DNA double-strand break repair after ionizing radiation in head and neck squamous carcinoma cells

We hypothesized that exosomes may promote survival by triggering DNA repair as it was shown that phosphorylation of critical DNA repair proteins is influenced by exosomes [21]. Our results showed that DNA repair was not influenced by exosomes at an early time point after irradiation (1 h), while increased DNA repair was found after incubation with exosomes from irradiated donor cells at later time points (6–10 h). As the increased DNA repair was equally detected for a 6 h incubation at which only a limited number of exosomes is associated to the cells and after a pre-incubation with exosomes we assume that a small amount of exosomes is sufficient to induce the observed effects. Different aspects of the impact of exosomes on the DNA repair were analyzed in two recent studies. One showed that an increased number of DNA repair foci was observed after transfer of exosomes from non-irradiated breast cancer cells to normal human primary mammary epithelial cells [21]. Using the comet-assay, Al-Mayah et al. on the other hand showed that exosomes increase the DNA damage of breast epithelial cancer cells [12]. However, both studies focus on the effect of exosomes on non-stressed cells while we provide data about the effects on radiation-stressed cells.

Several studies show that different cell lines exchange cellular components via exosomes suggesting that cell communication via exosomes is not cell type specific. Exosomes of colorectal cell lines for example deliver their content to hepatoma and lung cancer cell lines [3, 28]. In line with this, we verify that exosomes from BHY cells induce the same effects in the DSB repair in FaDu cells. This example is further evidence that exosomes are an intercellular communication tool and corroborates their already-suggested broad cell specificity. This is of great relevance for radiation therapy as the communication between irradiated and non-irradiated cells may be an important regulator of therapy outcome.

### 4.3 Radiation increases exosomal release and uptake in head and neck squamous carcinoma cells

In addition to the analysis of exosomal effects on recipient cells we focused on the exosomal release and uptake in the context of radiation. Irradiation increases the number of exosomes in the cell supernatant, suggesting that radiation augments the overall amount of exosome release. This is in accordance with studies describing radiation-increased exosome release in glioblastoma, prostate cancer and lung cancer cells [11, 29, 30]. Irradiation with the high dose of 9 Gy reduces the exosome release compared to 3 and 6 Gy irradiated cells. Possibly the enhanced damage increased the induction of cell death processes and counteracts the release of exosomes. Irradiation does not change the exosomal size, whereby the isolated exosomes from BHY cells with an average size of 111–124 nm are at the upper size limit.

We confirm the influence of radiation on the uptake of exosomes by using fluorescence-labeled exosomes. Fluorescence microscopy pictures visualized the attachment of clusters of exosomes to the cell membrane at an early time point followed by their internalization and distribution in the cytoplasm at later time points. FACS analysis further demonstrated a dose-dependent increased uptake of exosomes by irradiated recipient cells. We assume that irradiation induces the uptake of exosomes by recipient cells. This finding is in accordance with the increased uptake of exosomes by mesenchymal stem cells and glioblastoma cells upon irradiation through augmented CD29/CD81 complex formation [24]. An increased uptake of exosomes from irradiated donor cells as shown for glioblastoma cells is not detected in this study for head and neck cancer cells [11]. However, we cannot conclude if exosomes have to be internalized or if they induce the observed effects through the association to the cell membranes alone.

### 4.4 Exosome cargo increases resistance against tumor eliminating therapies

The development of therapy resistance is the limiting factor of cancer treatments. An exosome-conferred increase in drug resistance has been shown for several cell lines and compounds [16–19]. We demonstrate that exosomes from irradiated donor cells also increase radiation resistance and increase DNA repair in head and neck squamous carcinoma cells. A decreased α/β-ratio of the survival curve after transfer of exosomes also suggests an increase in the DNA repair capacity (S3 Table). However, exosomes from non-irradiated donor cells also produced a slight increase in radiation resistance, while they did not accelerate DNA double strand break repair it is obvious that also other pathways beside repair contribute to the increased survival. Basically exosome quantity and exosome cargo may contribute to the observed biological effects. But as the number of released exosomes and the biological effects do not correlate, we suggest that exosomal effects are mainly caused by a change in exosomal composition or cargo. Several investigations reveal that cellular stress can alter the exosomal RNA composition [10, 11, 22]. According to these findings, and based on the finding that RNase treatment abrogates the effects of exosomes on DNA repair, we suggest that exosomal RNA molecules (either attached to or included into exosomes) may trigger repair processes in recipient cells. This finding for extracellular RNA stands in line with our previous studies which showed that the expression of intracellular microRNA and long non-coding RNA supports survival of irradiated cells [4, 31, 32].

## 5 Conclusion

We have evaluated the role of exosomes in the response of head and neck cancer cells to radiation. Our results show that exosomes can serve as a communication tool in the acute radiation stress-response and confer protective signals to neighboring cells. We conclude that exosomes transmit prosurvival signals and therefore promote the tumorigenic and radioresistant phenotype of head and neck cancer cells. This study indicates a functional role for exosomes in the response of tumor cells to therapeutic radiation exposure and encourages that exosomes are useful targets to improve therapy strategies.

## Supporting Information

S1 Fig **(A)** BHY cells were categorized according to the foci number per cell (0–29). For each experiment the foci number of 60 BHY cells was determined 6 hours after irradiation with 2 Gy and transfer of BHY exosomes isolated 24 hours after irradiation with 0 and 6 Gy [n = 3]. **(B)** Relative number of 53BP1 foci in BHY cells 6, 8 and 10 hours after 2 Gy and transfer of BHY exosomes isolated 24 hours after irradiation [n_1_ (6 h control; 6 h EXO 0 Gy; 6 h EXO 6 Gy) = 6, n_2_ (6 h EXO 3 Gy; 6 h EXO 9 Gy; 8 h; 10 h) = 3, ± SD]. **(C)** BHY cells were pre-incubated with exosomes, irradiated 24 hours later and the number of 53BP1 foci was determined 6 hours after irradiation [n = 3, ± SD]. For all experiments the p-values calculated on control were considered to be significant if * p < 0.05 and highly significant ** if p < 0.01, while ^▲^ p < 0.05 and ^▲▲^ p < 0.01 indicate significant differences to EXO 0 Gy.
(TIFF)

S1 Table Authentication of BHY cell line. A short tandem repeat profile was obtained by PCR amplification of eight core short tandem repeat loci plus amelogenin for sex determination. Authentication of cells was performed by comparing the results with the online DMSZ Profile Database (www.dmsz.de). In the diagram the best fitting five cell lines of this alignment with the database are depicted. The authentication for BHY matches to 100%.
(XLS)

S2 Table Authentication of FaDu cell line. A short tandem repeat profile was obtained by PCR amplification of eight core short tandem repeat loci plus amelogenin for sex determination. Authentication of cells was performed by comparing the results with the online DMSZ Profile Database (www.dmsz.de). In the diagram the best fitting five cell lines of this alignment with the database are depicted. For the tested FaDu cells the best fitting database profile was obtained from FaDu cells with a 88.3% match.
(XLS)

S3 Table Clonogenic survival of BHY cells. Data were plotted on a semi-log scale and fitted to the linear quadratic equation SF = e^(-αD-βD^2)^. Parameters α and β were used to calculate the α/ β ratio, the inactivation dose for 37% survival (D37) and the surviving fraction at a dose of 2 Gy (SF2).
(XLS)

## Abbreviations

DMEM: Dulbecco’s modified Eagle’s medium
DSB: double-strand break
EXO: exosomes
HNSCC: head and neck squamous cell carcinoma

## References

[1] Al-Nedawi K, Meehan B, Micallef J, Lhotak V, May L, Guha A, et al Intercellular transfer of the oncogenic receptor EGFRvIII by microvesicles derived from tumour cells. Nature cell biology. 2008;10(5):619–24. 10.1038/ncb1725 . 18425114

[2] Crescitelli R, Lasser C, Szabo TG, Kittel A, Eldh M, Dianzani I, et al Distinct RNA profiles in subpopulations of extracellular vesicles: apoptotic bodies, microvesicles and exosomes. Journal of extracellular vesicles. 2013;2 10.3402/jev.v2i0.20677 24223256 PMC3823106

[3] Valadi H, Ekstrom K, Bossios A, Sjostrand M, Lee JJ, Lotvall JO. Exosome-mediated transfer of mRNAs and microRNAs is a novel mechanism of genetic exchange between cells. Nature cell biology. 2007;9(6):654–9. 10.1038/ncb1596 . 17486113

[4] O'Leary VB, Ovsepian SV, Carrascosa LG, Buske FA, Radulovic V, Niyazi M, et al PARTICLE, a Triplex-Forming Long ncRNA, Regulates Locus-Specific Methylation in Response to Low-Dose Irradiation. Cell reports. 2015 10.1016/j.celrep.2015.03.043 . 25900080

[5] Raposo G, Stoorvogel W. Extracellular vesicles: exosomes, microvesicles, and friends. The Journal of cell biology. 2013;200(4):373–83. 10.1083/jcb.201211138 23420871 PMC3575529

[6] Peinado H, Aleckovic M, Lavotshkin S, Matei I, Costa-Silva B, Moreno-Bueno G, et al Melanoma exosomes educate bone marrow progenitor cells toward a pro-metastatic phenotype through MET. Nature medicine. 2012;18(6):883–91. 10.1038/nm.2753 22635005 PMC3645291

[7] Mineo M, Garfield SH, Taverna S, Flugy A, De Leo G, Alessandro R, et al Exosomes released by K562 chronic myeloid leukemia cells promote angiogenesis in a Src-dependent fashion. Angiogenesis. 2012;15(1):33–45. 10.1007/s10456-011-9241-1 22203239 PMC3595015

[8] Wang J, Hendrix A, Hernot S, Lemaire M, De Bruyne E, Van Valckenborgh E, et al Bone marrow stromal cell-derived exosomes as communicators in drug resistance in multiple myeloma cells. Blood. 2014;124(4):555–66. 10.1182/blood-2014-03-562439 . 24928860

[9] Eldh M, Ekstrom K, Valadi H, Sjostrand M, Olsson B, Jernas M, et al Exosomes communicate protective messages during oxidative stress; possible role of exosomal shuttle RNA. PloS one. 2010;5(12):e15353 10.1371/journal.pone.0015353 21179422 PMC3003701

[10] King TL, Heesch CM, Clark CG, Kline DD, Hasser EM. Hypoxia activates nucleus tractus solitarii neurons projecting to the paraventricular nucleus of the hypothalamus. American journal of physiology Regulatory, integrative and comparative physiology. 2012;302(10):R1219–32. 10.1152/ajpregu.00028.2012 22403798 PMC3362152

[11] Arscott WT, Tandle AT, Zhao S, Shabason JE, Gordon IK, Schlaff CD, et al Ionizing Radiation and Glioblastoma Exosomes: Implications in Tumor Biology and Cell Migration. Translational Oncology. 2013;6(6):638–IN6. 10.1593/tlo.13640 24466366 PMC3890698

[12] Al-Mayah AH, Irons SL, Pink RC, Carter DR, Kadhim MA. Possible role of exosomes containing RNA in mediating nontargeted effect of ionizing radiation. Radiation research. 2012;177(5):539–45. . 22612287 10.1667/rr2868.1

[13] Al-Mayah A, Bright S, Chapman K, Irons S, Luo P, Carter D, et al The non-targeted effects of radiation are perpetuated by exosomes. Mutation research. 2015;772:38–45. 10.1016/j.mrfmmm.2014.12.007 . 25772109

[14] Jella KK, Rani S, O'Driscoll L, McClean B, Byrne HJ, Lyng FM. Exosomes Are Involved in Mediating Radiation Induced Bystander Signaling in Human Keratinocyte Cells. Radiation research. 2014 10.1667/RR13337.1 . 24502353

[15] Adelstein DJ, Li Y, Adams GL, Wagner H Jr, Kish JA, Ensley JF, et al An intergroup phase III comparison of standard radiation therapy and two schedules of concurrent chemoradiotherapy in patients with unresectable squamous cell head and neck cancer. Journal of clinical oncology: official journal of the American Society of Clinical Oncology. 2003;21(1):92–8. . 12506176 10.1200/JCO.2003.01.008

[16] Corcoran C, Rani S, O'Brien K, O'Neill A, Prencipe M, Sheikh R, et al Docetaxel-resistance in prostate cancer: evaluating associated phenotypic changes and potential for resistance transfer via exosomes. PloS one. 2012;7(12):e50999 10.1371/journal.pone.0050999 23251413 PMC3519481

[17] Chen WX, Cai YQ, Lv MM, Chen L, Zhong SL, Ma TF, et al Exosomes from docetaxel-resistant breast cancer cells alter chemosensitivity by delivering microRNAs. Tumour biology: the journal of the International Society for Oncodevelopmental Biology and Medicine. 2014;35(10):9649–59. 10.1007/s13277-014-2242-0 . 24969560

[18] Battke C, Ruiss R, Welsch U, Wimberger P, Lang S, Jochum S, et al Tumour exosomes inhibit binding of tumour-reactive antibodies to tumour cells and reduce ADCC. Cancer immunology, immunotherapy: CII. 2011;60(5):639–48. 10.1007/s00262-011-0979-5 . 21293856 PMC11029199

[19] Ciravolo V, Huber V, Ghedini GC, Venturelli E, Bianchi F, Campiglio M, et al Potential role of HER2-overexpressing exosomes in countering trastuzumab-based therapy. Journal of cellular physiology. 2012;227(2):658–67. 10.1002/jcp.22773 . 21465472

[20] Théry C, Amigorena S, Raposo G, Clayton A. Isolation and Characterization of Exosomes from Cell Culture Supernatants and Biological Fluids Current Protocols in Cell Biology: John Wiley & Sons, Inc; 2001. 10.1002/0471143030.cb0322s30 18228490

[21] Dutta S, Warshall C, Bandyopadhyay C, Dutta D, Chandran B. Interactions between Exosomes from Breast Cancer Cells and Primary Mammary Epithelial Cells Leads to Generation of Reactive Oxygen Species Which Induce DNA Damage Response, Stabilization of p53 and Autophagy in Epithelial Cells. PloS one. 2014;9(5):e97580 10.1371/journal.pone.0097580 24831807 PMC4022578

[22] de Jong OG, Verhaar MC, Chen Y, Vader P, Gremmels H, Posthuma G, et al Cellular stress conditions are reflected in the protein and RNA content of endothelial cell-derived exosomes. Journal of extracellular vesicles. 2012;1 10.3402/jev.v1i0.18396 24009886 PMC3760650

[23] Park JE, Tan HS, Datta A, Lai RC, Zhang H, Meng W, et al Hypoxic tumor cell modulates its microenvironment to enhance angiogenic and metastatic potential by secretion of proteins and exosomes. Molecular & cellular proteomics: MCP. 2010;9(6):1085–99. 10.1074/mcp.M900381-MCP200 20124223 PMC2877972

[24] Hazawa M, Tomiyama K, Saotome-Nakamura A, Obara C, Yasuda T, Gotoh T, et al Radiation increases the cellular uptake of exosomes through CD29/CD81 complex formation. Biochemical and biophysical research communications. 2014;446(4):1165–71. 10.1016/j.bbrc.2014.03.067 . 24667602

[25] Yang L, Wu XH, Wang D, Luo CL, Chen LX. Bladder cancer cell-derived exosomes inhibit tumor cell apoptosis and induce cell proliferation in vitro. Molecular medicine reports. 2013;8(4):1272–8. 10.3892/mmr.2013.1634 . 23969721

[26] Raimondo S, Saieva L, Corrado C, Fontana S, Flugy A, Rizzo A, et al Chronic myeloid leukemia-derived exosomes promote tumor growth through an autocrine mechanism. Cell communication and signaling: CCS. 2015;13(1):8 10.1186/s12964-015-0086-x 25644060 PMC4320527

[27] Xiao H, Lasser C, Shelke GV, Wang J, Radinger M, Lunavat TR, et al Mast cell exosomes promote lung adenocarcinoma cell proliferation—role of KIT-stem cell factor signaling. Cell communication and signaling: CCS. 2014;12(1):64 10.1186/s12964-014-0064-8 25311367 PMC4206705

[28] Chiba M, Kimura M, Asari S. Exosomes secreted from human colorectal cancer cell lines contain mRNAs, microRNAs and natural antisense RNAs, that can transfer into the human hepatoma HepG2 and lung cancer A549 cell lines. Oncology reports. 2012;28(5):1551–8. 10.3892/or.2012.1967 22895844 PMC3583404

[29] Lehmann BD, Paine MS, Brooks AM, McCubrey JA, Renegar RH, Wang R, et al Senescence-associated exosome release from human prostate cancer cells. Cancer research. 2008;68(19):7864–71. 10.1158/0008-5472.CAN-07-6538 18829542 PMC3845029

[30] Wysoczynski M, Ratajczak MZ. Lung cancer secreted microvesicles: underappreciated modulators of microenvironment in expanding tumors. International journal of cancer Journal international du cancer. 2009;125(7):1595–603. 10.1002/ijc.24479 19462451 PMC2769262

[31] Kraemer A, Anastasov N, Angermeier M, Winkler K, Atkinson MJ, Moertl S. MicroRNA-Mediated Processes are Essential for the Cellular Radiation Response. Radiation research. 2011;176(5):575–86. 10.1667/RR2638.1 21854212

[32] Kraemer A, Barjaktarovic Z, Sarioglu H, Winkler K, Eckardt-Schupp F, Tapio S, et al Cell survival following radiation exposure requires miR-525-3p mediated suppression of ARRB1 and TXN1. PloS one. 2013;8(10):e77484 10.1371/journal.pone.0077484 24147004 PMC3797807

