# Supplementary figures and images for: Exosomes Derived from Squamous Head and Neck Cancer Promote Cell Survival after Ionizing Radiation
Source: PLoS One. 2016 Mar 23;11(3):e0152213. doi: 10.1371/journal.pone.0152213 (PMC4805173; doi:10.1371/journal.pone.0152213)

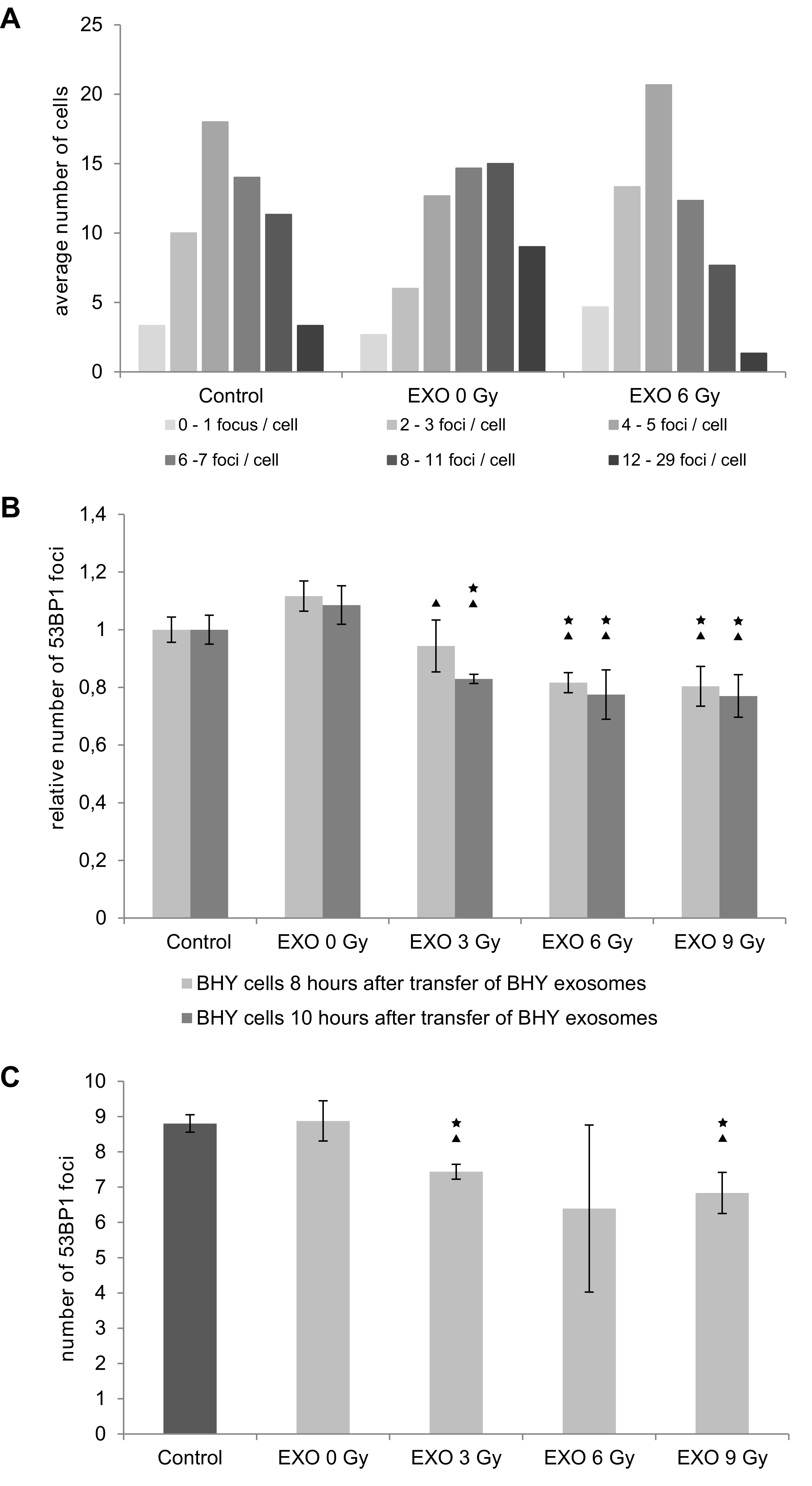

Supplement: S1 Fig — (A) BHY cells were categorized according to the foci number per cell (0–29). For each experiment the foci number of 60 BHY cells was determined 6 hours after irradiation with 2 Gy and transfer of BHY exosomes isolated 24 hours after irradiation with 0 and 6 Gy [n = 3]. (B) Relative number of 53BP1 foci in BHY cells 6, 8 and 10 hours after 2 Gy and transfer of BHY exosomes isolated 24 hours after irradiation [n1 (6 h control; 6 h EXO 0 Gy; 6 h EXO 6 Gy) = 6, n2 (6 h EXO 3 Gy; 6 h EXO 9 Gy; 8 h; 10 h) = 3, ± SD]. (C) BHY cells were pre-incubated with exosomes, irradiated 24 hours later and the number of 53BP1 foci was determined 6 hours after irradiation [n = 3, ± SD]. For all experiments the p-values calculated on control were considered to be significant if * p < 0.05 and highly significant ** if p < 0.01, while ▲ p < 0.05 and ▲▲ p < 0.01 indicate significant differences to EXO 0 Gy. (TIFF) [file pone.0152213.s001.tiff]
